# Phylogenetic Position and Replication Kinetics of *Heliothis virescens* Ascovirus 3h (HvAV-3h) Isolated from *Spodoptera exigua*

**DOI:** 10.1371/journal.pone.0040225

**Published:** 2012-07-05

**Authors:** Guo-Hua Huang, Tyler A. Garretson, Xin-Hua Cheng, Maria S. Holztrager, Shun-Ji Li, Xing Wang, Xiao-Wen Cheng

**Affiliations:** 1 Hunan Provincial Key Laboratory for Biology and Control of Plant Diseases and Insect Pests, Hunan Agricultural University, Changsha, Hunan, China; 2 . Department of Microbiology, Miami University, Oxford, Ohio, United States of America; Central China Normal University, China

## Abstract

Insect-specific ascoviruses with a circular genome are distributed in the USA, France, Australia and Indonesia. Here, we report the first ascovirus isolation from *Spodoptera exigua* in Hunan, China. DNA-DNA hybridization to published ascoviruses demonstrated that the new China ascovirus isolate is a variant of *Heliothis virescens ascovirus 3a* (HvAV-3a), thus named HvAV-3h. We investigated the phylogenetic position, cell infection, vesicle production and viral DNA replication kinetics of HvAV-3h, as well as its host-ranges. The major capsid protein (MCP) gene and the delta DNA polymerase (DNA po1) gene of HvAV-3h were sequenced and compared with the available ascovirus isolates for phylogenetic analysis. This shows a close relationship with HvAV-3g, originally isolated from Indonesia, HvAV-3e from Australia and HvAV-3c from United States. HvAV-3h infection induced vesicle production in the SeE1 cells derived from *S. exigua* and Sf9 cells derived from *S. frugiperda*, resulting in more vesicles generated in Sf9 than SeE1. Viral DNA replication kinetics of HvAV-3h also demonstrated a difference between the two cell lines tested. HvAV-3h could readily infect three important insect pests *Helicoverpa armigera* (Hübner), *Spodoptera exigua* (Hübner) and *Spodoptera litura* (Fabricius) from two genera in different subfamilies with high mortalities.

## Introduction

Among viruses that infect insects, ascovirus is a recently discovered group of viruses with a circular double-stranded DNA genome ranging in size of 100-186 kbp [1], [2], [3], [4], [5]. Unlike the well-studied insect-specific baculovirus that often causes larvae to liquefy, making it relative easy to identify, the apparent scarcity of ascoviruses is likely due to the poor manifestation of infection symptoms in insects [6]. Another fact that may also contribute to the late discovery of ascoviruses is the poor *per os* infectivity to insects resulting in their scarcity in nature [2], [7]. Therefore, the primary route of larvae to larvae transmission of ascoviruses in the natural environment is through the oviposition by wasp in larvae [8], [9]. However, in the parasitized larvae that are infected by ascovirus, the larvae of parasitic wasps could not develop properly and eventually die of ascovirus infection [9]. Despite the difficulties in ascovirus isolation, ascoviruses were first discovered in the Southeastern and Western United States in the late 1970’s [10], [11], [12], [13]. Other ascoviruses have been reported in France, Australia and Indonesia [14], [15], [16].

Traditionally, ascovirus had been named after the host from which the ascovirus is isolated [3]. As more ascoviruses are isolated, naming ascoviruses after the host insects become confusing due to the fact that some ascoviruses can infect a wide range of lepidopteran larvae [17]. It wasn’t until 1999 before the family *Ascoviridae* was established by the International Committee on Virus Taxonomy (ICTV) with four recognized ascovirus species based virion morphology, host range and DNA-DNA hybridization [18]. The four recognized ascovirus species are *Spodoptera frugiperda ascovirus 1a* (SfAV-1a) which is also the type species of the *Ascoviridae*, *Trichoplusia ni ascovirus 2a* (TnAV-2a), *Heliothis virescens ascovirus 3a* (HvAV-3a) and *Diadromus puchellus ascovirus 4a* (DpAV-4a) isolated from the parasitic ichneumonid wasp *Diadromus puchellus* [18]. More recently, as information becomes available, three new ascovirus species have been proposed for future discussion [19]. At the same time, the previously recognized DpAV-4a is believed to have evolved from a different lineage than other lepidopteran ascoviruses based on core gene phylogenetic analysis [20]. Prior to the publication of the DpAV-4a genome, three ascovirus genomes, TnAv-2c, SfAV-1a and HvAV-3e, had been sequenced and reported [4], [5], [21]. In each of the three lepidopteran ascovirus species, several isolates have been reported with HvAV-3a having the most variants reported [19]. HvAV-3a is also the only ascovirus species that has been reported in regions outside of America, where the earlier SfAV-1a and TnAV-2a were isolated.

The three ascovirus species show markedly different host ranges in infecting lepidopteran larvae. SfAV-1a causes mortality only in the *Spodoptera* spp and replicates solely in the fat body tissue. Whereas, TnAV-2a and HvAV-3a, which infect many lepidopteran hosts, replicate in the epidermis, tracheal epithelilum and fat body tissue [2], [17]. During larval infection, ascoviruses partition the infected cells to form virion containing vesicles that are released into the hemolymph [17]. Accumulation of virion containing vesicles in the hemolymph results in color change from clear to milky white [2]. The partition or vesicle formation has been linked to the expression of viral caspase gene [22].

Even though ascoviruses have been reported in several regions of the world, no ascoviruses have yet been isolated and reported in China. Various efforts had been extended to isolate ascoviruses in this large country in the past 10 or so years, but have been unsuccessful. In 2011, the first ascovirus was found in the larvae of *Spodoptera exigua* in a cotton field in Changsha, Hunan Province, China. In this report, we confirmed that the ascovirus isolated from *S. exigua* belongs to the species *Heliothis virescens ascovirus 3a* based on DNA-DNA dot-blot, Southern hybridization and partial genome sequencing analysis, and thus named HvAV-3h. Phylogenetic analysis using the conserved region of ascovirus delta DNA polymerase (DNA pol) and the partial major capsid protein (MCP) protein sequences of HvAV-3h with the 17 reported ascovirus isolates showed that it forms a cluster with other HvAV-3a variants and some TnAV-2 isolates [23]. We further compared the replication kinetics in different cell lines and host range in insects.

## Results

### Isolation of HvAV-3h

**Figure 1:**
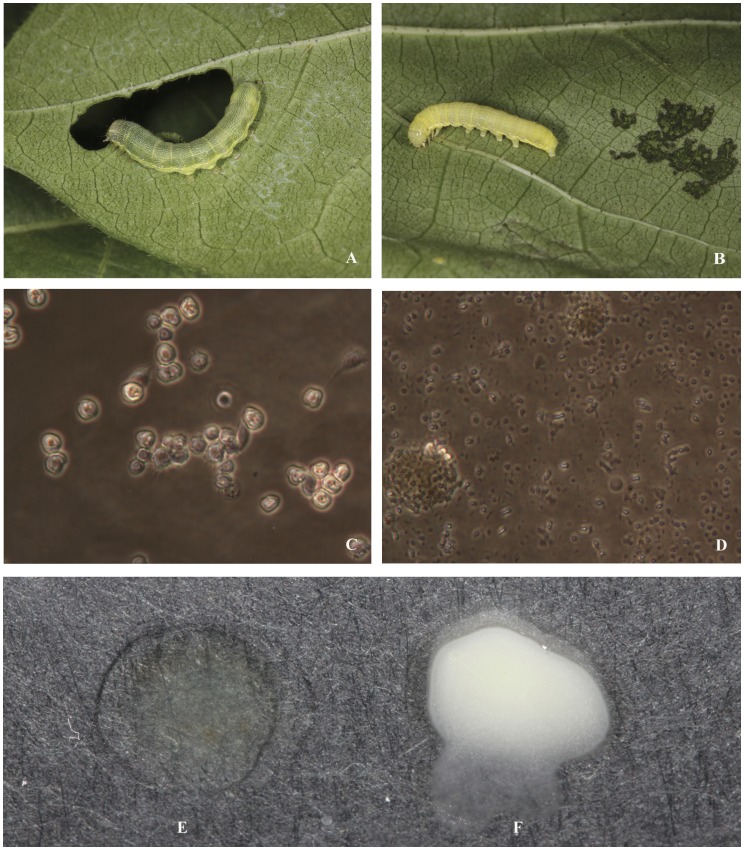
Comparison of healthy and ascovirus infected larvae of *Spodoptera exigua*. **A**, healthy larva showing greenish color and being able to chew hole on cotton leaf; **B**, infected larva showing yellowish color and being only able to scrap on surface of leaf; **C**, healthy hemolymph showing intact cells under an inverted microscope (400 x); **D**, ascovirus infected hemolymph showing vesicle production under an inverted microscope (400 x); **E**, hemolymph color of healthy larva; **F**, hemolymph color of ascovirus infected larvae.

Collection of lepidopteran larvae for isolating ascovirus started in June, 2011 during the growing season of cotton, but no ascovirus had been found. Collection of lepidopteran larvae continued in August and three thousands larvae of *S. exigua* were collected and reared individually in the lab for observation of larvae with extended larval stage. Among the 3,000 larvae collected, eight larvae showed stunt larval growth. Further examination of larval hemolymph revealed that only one larva showed white milky color suggesting ascovirus infection. The hemolymph from this larva containing suspected ascovirus was used for the subsequent studies in this report. In the field, the *S. exigua* larva suspecting of ascovirus infection showed yellowish appearance and was found near an area of web appearance at the underside of the cotton leaf. This was most likely caused by surface scraping by the ascovirus infected larva in contrast to hole-making of leaves chewed by uninfected *S. exigua* larvae (Fig. 1A and B).

To confirm that this *S. exigua* larva was infected by ascovirus, the hemolymph was used to inoculate healthy *S. exigua* larvae. Inoculated larvae showed typical ascovirus infection with reduced larval growth and extended larval stages. When the hemolymph of healthy and infected larvae was bled for examination under the microscope, distinguishable cells, possible hemocytes, from the healthy larval hemolymph were detected, whereas small reflective bodies, presumably the vesicles, were found in the hemolymph of infected larvae (Fig.1C and D). It is interesting to note that no cell was found in the hemolymph of infected larvae suggesting that the possible hemocytes were also infected leading to partitioning of these cells (Fig. 1C and D). When more hemolymph, both healthy and infected larvae, were applied on a glass slide, the hemolymph of healthy larva were primarily clear but the infected showed a milky-white color (Fig. 1E and F).

**Figure 2:**
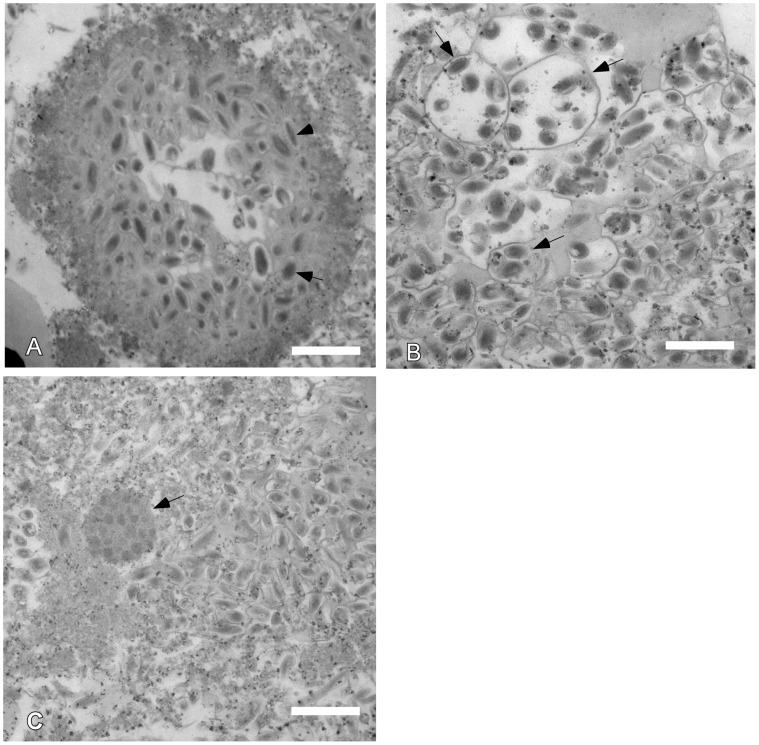
Transmission electron microscopy of HvAV-3h. Hemolymph of *S. exigua* larvae infected with HvAV-3h was harvested and processed for TEM. **A**, a virion containing vesicle showing ovoidal (arrow) or bacilliform (arrow head) shape of HvAV-3h virions. **B**, a vesicle that contains virion containing vacuoles (arrow). **C**, a vesicle that contains a occlusion body (arrow). Scale bar, 500 nm.

Transmission electron microscopy (TEM) of the milky-white hemolymph showed virion containing vesicles similar to HvAV-3g (SeAV-5a) [24]. The virions were either of ovoidal or bacilliform shape (Fig. 2A). The virion measured 300±22 × 130±95 nm. In some vesicles, 2 to 7 virions were found in large vacuoles (Fig. 2B). Occlusion bodies containing dark-staining bodies were also found in some vesicles (Fig. 2C). However, these dark-staining bodies didn’t appear like either baculovirus or ascovirus because they were without an envelope (Fig. 2C). No other viruses including baculoviruses were found during TEM suggesting the pure nature of this ascovirus.

#### Viral DNA-DNA dot blot, DNA restriction profile and Southern hybridization

**Figure 3:**
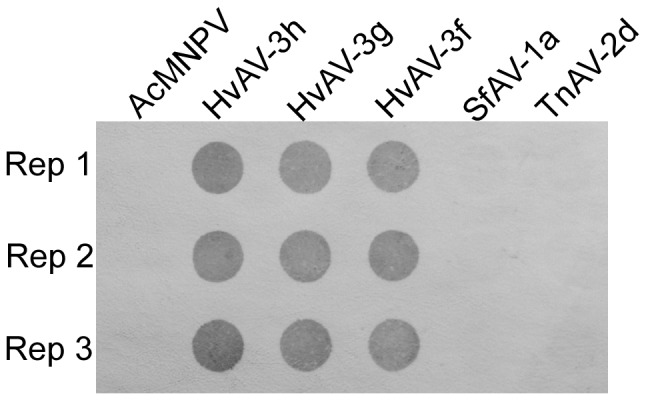
Dot blot DNA-DNA hybridization between HvAV-3h and other viruses (AcMNPV, HvAV-3h, HvAV-3g, HvAV-3f, SfAV-1a, TnAV-2d). Viral DNA (100 ng) each was blotted to a nylon membrane and probed with HvAV-3h DNA labeled with biotin and detected with the streptavidin/biotinylated alkaline phosphatase system. Rep, replication.

In order to identify this newly isolated ascovirus at the species level, viral DNA was extracted and purified from virions. DNA-DNA dot bot hybridization, restriction fragment length polymorphism (RFLP) and Southern hybridization studies were performed with other reported ascovirus species. In the dot blot hybridization analysis, the probe, containing the new ascovirus HvAV-3h, hybridized strongly to its homologous genomic DNA, but also to HvAV-3g (SeAV-5a) and HvAV-3f. No discernible hybridization occurred with the negative control AcMNPV and the other two ascovirus species, SfAV-1a and TnAV-2d (Fig. 3). A more detailed RFLP and Southern hybridization were used to probe specific regional DNA hybridization.

**Figure 4:**
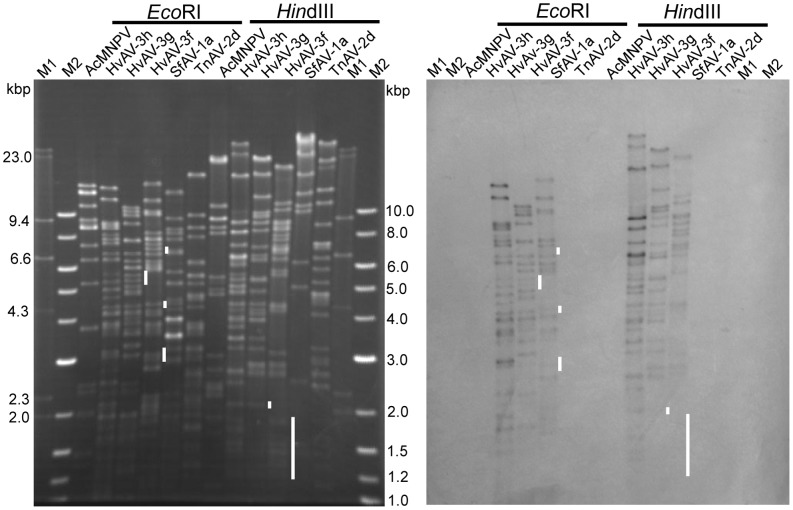
RFLP and Southern Hybridization analysis. A , RFLP of AcMNPV, HvAV-3h, HvAV-3g, HvAV-3f, SfAV-1a, TnAV-2d were digested individually with *Eco*R1 and *Hin*dIII and separated by 0.7% agarose gel electrophoresis; **B**, Southern hybridization with the HvAV-3h biotin labeled probe. M1, λ/HindIII DNA size marker. M2, 1 kb DNA ladder. Vertical bars indicate DNA fragments not showing hybridization to the HvAV-3h probe.

Using the *Eco*RI and *Hin*dIII restriction fragments of HvAV-3g genomic DNA, we estimated the genome size of HvAV-3h to be about 164 kbp (Fig. 4A). The RFLP showed some co-migrating *Eco*RI and *Hin*dIII fragments among HvAV-3h, HvAV-3g and HvAV-3f. However, this does not provide significant genetic homology information among the three HvAV-3a variants. Southern hybridization confirmed that the new ascovirus isolated from China was indeed HvAV-3h by demonstrating strong hybridization to HvAV-3g and HvAV-3f, but not to the negative control AcMNPV and two other ascovirus species SfAV-1a and TnAV-2d (Fig. 4B). However, there were specific regions or *Eco*RI and *Hin*dIII fragments of HvAV-3g and HvAV-3f that showed no or weak hybridization to the HvAV-3h probe (Fig. 4B). This suggests that the new isolate HvAV-3h is not identical to HvAV-3g and HvAV-3f.

### Cell Infection and Viral DNA Replication Kinetics

**Figure 5:**
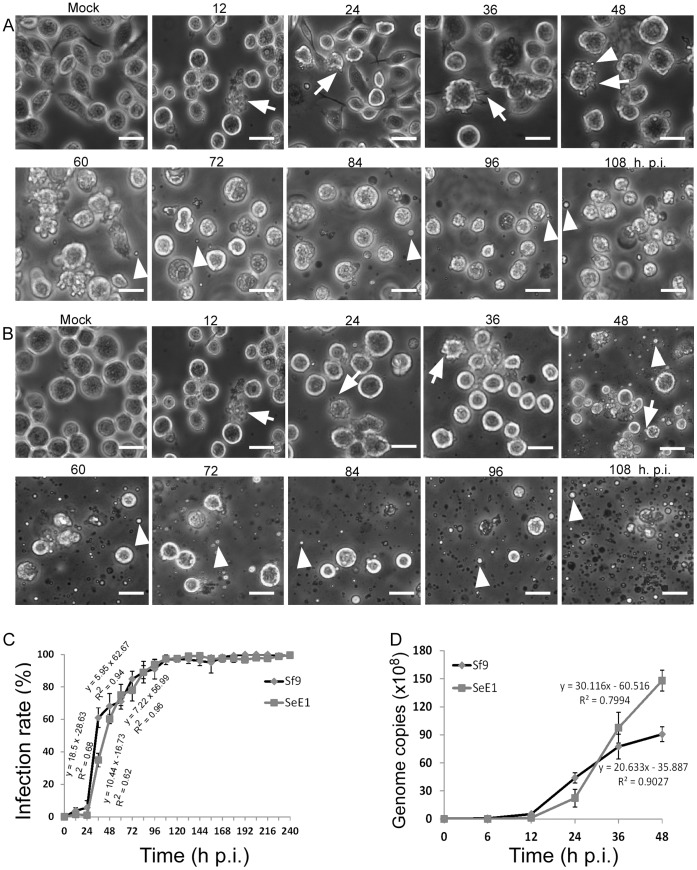
Comparison of HvAV-3h infection and DNA replication kinetics in SeE1 and Sf9 cells. A , SeE1 cells infected with HvAV-3h showing cytopathology at different time post infection. Arrows point to cells showing infection. Arrow heads point to vesicles release from infected cells, **B**, Sf9 cells infected with HvAV-3h showing cytopathology at different time post infection. Arrows point to cells showing infection. Arrow heads point to vesicles release from infected cells. Bar, 20 µm. **C**, comparison of HvAV-3h infection kinetics in SeE1 and Sf9 cells. Vertical bars denote standard deviation calculated from four independent infections. **D**, Comparison of HvAV-3h genome DNA replication kinetics. Vertical bars denote standard deviation calculated from three independent infections.

Since HvAV-3h is able to cleave and partition *S. exigua* larval cells to produce vesicles that accumulated to produce opaque white hemolymph (Fig. 1D and F), we sought to examine if it would also cleave and partition SeEI and Sf9 cells to produce vesicles typical of ascovirus infection. Using the same virion inoculum purified from the hemolymph of *S. exigua* larvae infected with HvAV-3h, infection rates based on the cytopathic effects induced by viral infection was monitored through 240 h post infection (p.i.). The cytopathic effects included blebbing of cytoplasmic membranes and projections from the cell, distortions of the cell when compared with mock infected cells. Infection was discernible at 12 h p.i. in some cells of both SeE1 and Sf9 cell lines when both cells showed cell cytopathic effects (Fig. 5A and B). However, long finger-like protrusions of cytoplasmic membranes were more prevalent in SeE1 cells (Fig. 5A-36 and A-48) than in Sf9 cells that produced shorter cytoplasmic membrane protrusions (Fig. 5B-36) at 36 and 48 h p.i. Vesicle production was detected in both SeE1 and Sf9 cells infected with HvAV-3h at 48 h p.i. with more production in Sf9 than in SeE1 cells (Fig. 5A-48 and Fig. 5 B-48). There was a major difference in vesicle production between the two cell lines starting at 48 h p.i. with continued increased production in Sf9 cells, but this rise could not be detected in SeE1 cells (Fig. 5A 48-108 and Fig. 5B 48-108). No cytopathic effects and vesicle production were found in the mock infected cells of both cell lines. At the end of the experiment (240 h p.i.), almost all the SeE1 cells were infected as showing distortion of the cells with some vesicle production similar to that at 108 h p.i. (Figure S1). However, most of the Sf9 cells were cleaved to form vesicles (Figure S1).

**Figure 6:**
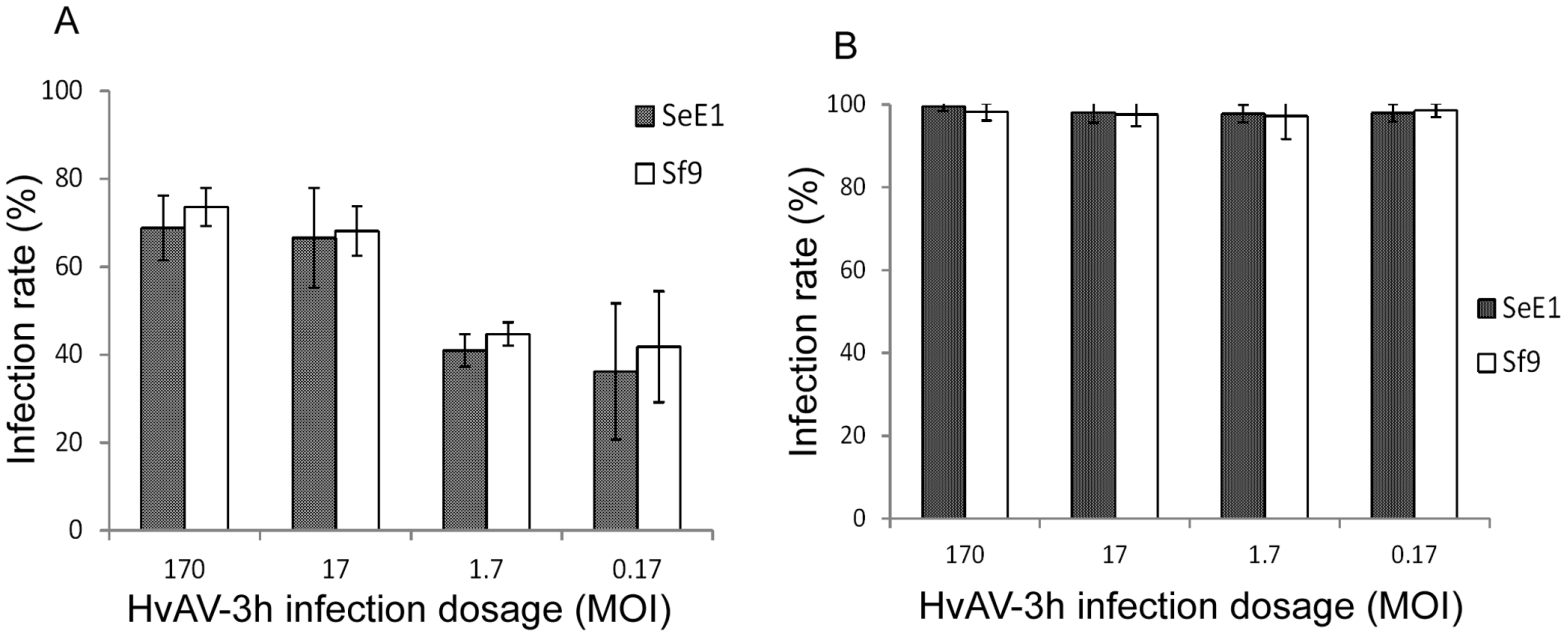
Dose response of SeE1 and Sf9 cells to HvAV-3h infection. SeE1 and Sf9 cells were infected separately with different multiplicity of infection (MOI) of HvAV-3h. Infection rates were calculated from four independent random pictures taken every 12 h p.i. until 240 h p.i. **A**, comparison of infection rates of different HvAV-3h MOI infection at 48 h p. i. **B**, comparison of infection rates of different HvAV-3h MOI infection at 108 h p. i. Vertical bars denote standard deviation of four independent infection rates.

There was a clear dose response of both cell lines to HvAV-3h infection. As MOI decreased, the number of cells showing cytopathic effects also decreased (Fig. 6A). Difference in infection rate of both cell lines was only found at early infection times, such as 48 h p.i. As infection progressed, both low and high MOI infections in both cell lines showed similar high infection rates close to 100% at 108 h p.i. (Fig. 6B). This suggests that HvAV-3h buds out of infected SeE1 and Sf9 cells during early times (i.e. 48 h p.i.) at lower MOIs, such as 1.7 and 0.17, and secondarily infects the other uninfected cells. We further investigated HvAV-3h cell infection kinetics.

As observed, after infection with the same HvAv-3h inoculum the Sf9 cells displayed cytopathic effects faster than SeE1 cells before 36 h p. i. (Fig. 5C). Even though the development of infection in SeE1 cells was slower than that in Sf9 cells, at 48 h p.i. infection rates in both cell lines reached about 70% and infection rates continued to increase until 108 h p.i when the infection rates for both cell lines reached almost 100%. The infection kinetics were similar between 48 to 108 h p.i. in both cell lines (Fig. 5C).

To investigate the DNA replication kinetics of HvAV-3h in SeE1and Sf9, cells were infected and DNA was extracted at different time points p. i. and the viral DNA copies were estimated by real-time qPCR. DNA replication of HvAV-3h in both cell lines were detected at 12 h p.i. which correlated with the cytopathic effect seen in cell culture infection at 12 h p.i. (Fig. 5A and B). The HvAV-3h replicated faster in SeE1 cells than in Sf9 cells, as supported by the larger slope value (30.116) in SeE1 cells than that in Sf9 cells (20.633) (Fig. 5D). HvAV-3h replicated more steadily in Sf9 cells than in SeE1 cells. Even though HvAV-3h replicated slower in SeE1 cells than Sf9 cells within the first 24 h p.i., it replicated faster in SeE1 cells than in Sf9 cells after 24 h p.i. (Fig. 5D).

### Host Range

**Figure 7:**
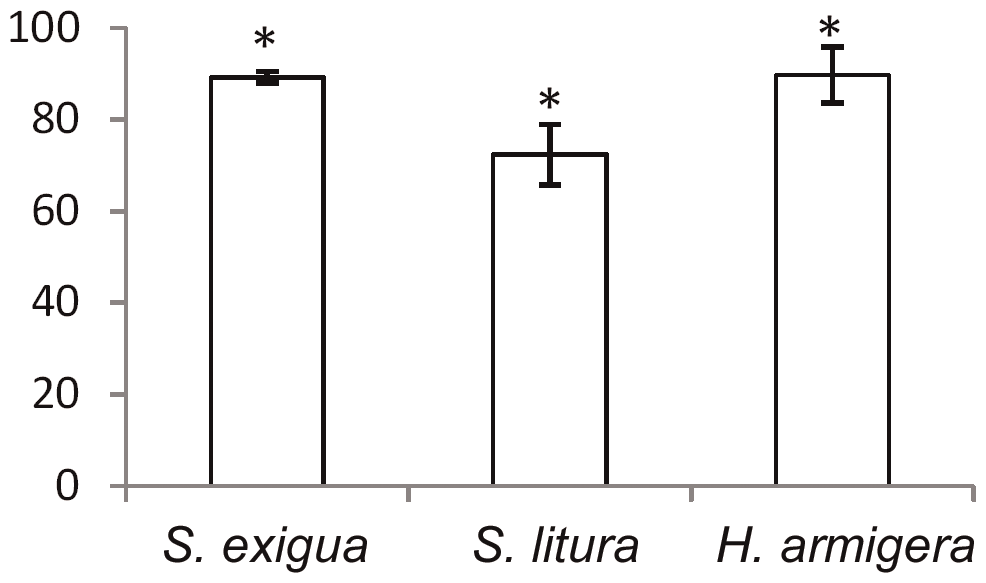
Comparison of HvAV-3h infectivity to insects. Thirty third instar larvae of each *H. armigera*, *S. exigua* and *S. litura* species were injected with HvAV-3h virions from hemolymph of a *S. exigua* larva infected with HvAV-3h. Control insects were injected with hemolymph from a healthy *S. exigua* larva. The mortality was recorded. The corrected mortality was used for the statistical test by ANOVA. *indicates no significant difference.

To investigate the host-range of HvAV-3h, larvae of three local noctuid pest species were infected by pinning the proleg of larvae with an insect pin contaminated with HvAV-3h. Typical ascovirus infection with development of yellowish larval color, reduced feeding and retarded growth was observed in all the three larval species. Mortality started at 13 day p.i. and continued to increase. At the end of the *in vivo* infection assay, the corrected mortalities of *H. armigera*, *S. exigua* and *S. litura* infected by HvAV-3h are 89.263±1.206%, 72.373±6.624% and 89.765±6.204%, respectively (Fig. 7). Based on ANOVA of the corrected mortalities, no statistically significant difference was found among those species infected with HvAV-3h (F1, 8 = 3.50723; P = 0.09799).

### Phylogenetic Analysis

**Figure 8:**
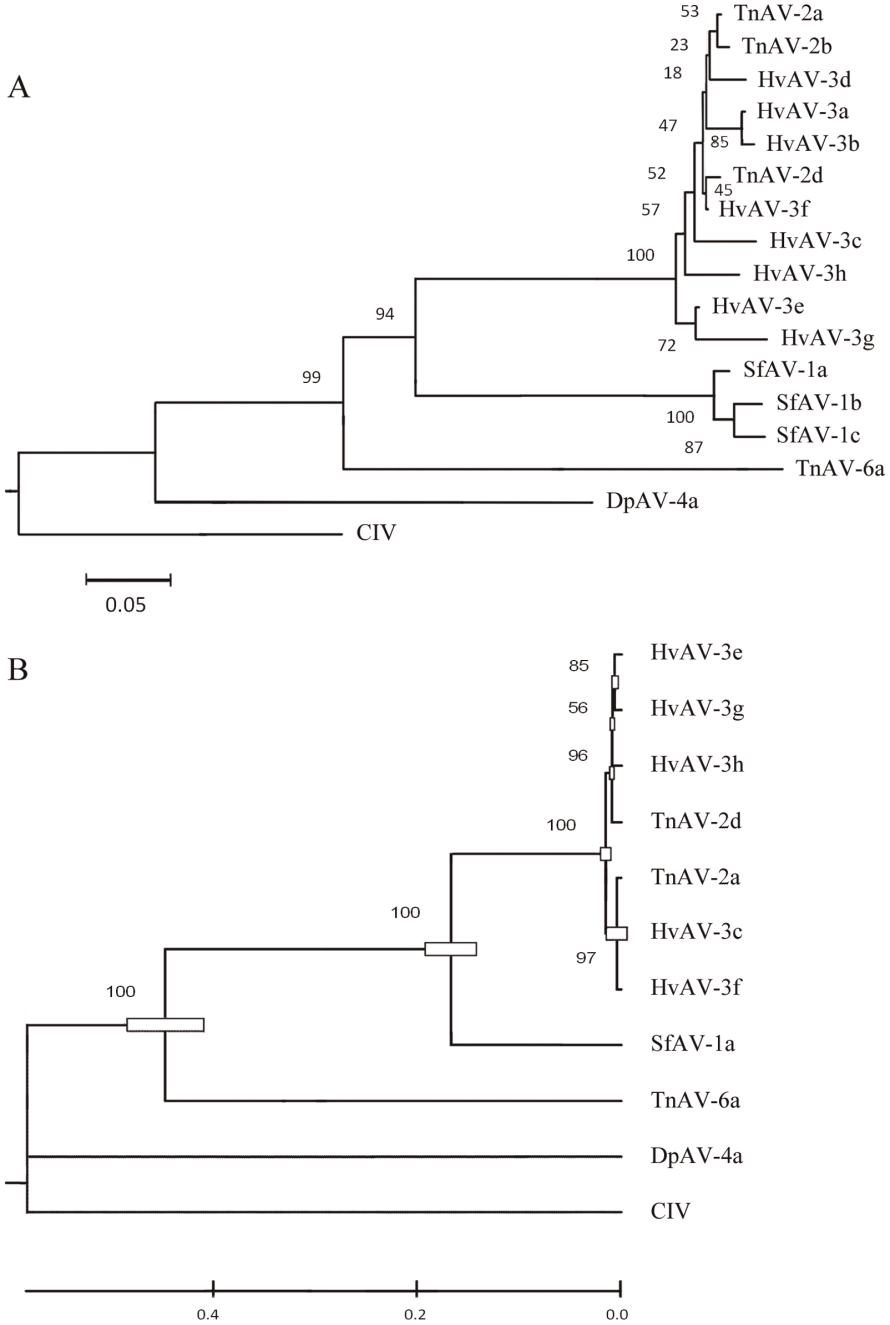
Phylogenetic analysis based on the combined protein sequences of the major capsid protein (MCP) and DNA polymerase (DNA Pol) genes. **A**, a NJ tree, numbers adjacent to branches are supporting values; **B**, a strict consensus maximum-likelihood tree, numbers adjacent to branches are supporting values. Chilo iridescent virus is used as an out-group in the phylogenetic analysis.

Conserved 205 amino acid fragments of the protein sequences of the delta polymerase (*po1*) gene from all of the named ascoviruses isolates (Table 1) were used to perform alignment, in order to determine the relationship of HvAV-3h to other ascoviruses. The NJ tree based on p-distance model showed that the isolate TnAV-6a formed a single species. The isolates SfAV-1a, SfAV-1b and SfAV-1c clustered as a monophyly supported by the bootstrap value of 100 belonging to the species SfAV-1a in. At the same time SfAV-1b and SfAV-1c formed a monophyly supported by the bootstrap value of 87. The isolates HvAV-3a, HvAV-3b, HvAV-3c, HvAV-3d, HvAV-3e, HvAV-3f, HvAV-3g, HvAV-3h, TnAV-2a, TnAV-2b and TnAV-2d are the monophyly supported by the bootstrap value of 100 but the relationship of HvAV-3h to other ascoviruses was unclear due to low bootstrap values (Fig. 8A). The combined protein sequences of *mcp* and DNA *po1* were used with the aligned length of 780 amino acids for phylogenetic analysis to understand the phylogenetic position of HvAV-3h. The best-fit model selected by Prottest 3.63 is WAG+G model with the lowest Bayesian Information Criterion (BIC) scores 11461.273 (-*lnL* = 5644.795). The MP bootstrap consensus tree shows largely similar topology to the ME tree and NJ tree, and the ML tree (Fig. 8B) also shows the same topology as the MrBayes tree. In these two trees, the monophyly of the family *Ascoviridae*, consisting of the species TnAV-6a, SfAV-1a, TnAV-2a and HvAV-3a, is well supported with the high bootstrap values using different analysis methods (100, 100, 100 and 98% in ML, ME, NJ and MP, respectively) and Bayesian posterior probability of 1.00 in MrBayes analysis (Fig. 8B). Comparison of HvAV-3h with the HvAV-3g, HvAV-3e and TnAV-2a isolates showed a close relationship supported by the high bootstrap values and Bayesian posterior probability. However, the phylogenetic position of DpAV-4a is still not clear.

**Table 1:** Information of the sequence data used in this study.

Species	Strains	Abbre-viations	Genes	Accession numbersNucleoid/Protein	References
*Chilo iridescent virus*	Chilo iridescent virus type 6	CIV	po1 for DNA Polymerase	AF303741/CAC19195	[48]
			Major capsid protein (MCP)	AF303741/Q05815	
*Diadromus puchellus* *ascovirus 4a*	Diadromus puchellus ascovirus 4a	DpAV-4a	po1 for DNA Polymerase	CU469068/CAC19127	[20]
			Major capsid protein (MCP)	CU469068/CAC84483	
*Trichoplusia ni ascovirus 6a*	Trichoplusia ni ascovirus 6a (2005)	TnAV-6a	po1 for DNA Polymerase	DQ517337/AAY43137	[5]
			Major capsid protein (MCP)	DQ517337/AAY43138	
*Spodoptera frugiperda* *ascovirus 1a*	Spodoptera frugiperda ascovirus 1a	SfAV-1a	po1 for DNA Polymerase	AM398843/AAC54632	[21]
			Major capsid protein (MCP)	AM398843/Q8JJY5	
	Spodoptera frugiperda ascovirus 1b	SfAV-1b	po1 for DNA Polymerase	AJ279823/translate	[49]
	Spodoptera frugiperda ascovirus 1c	SfAV-1c	po1 for DNA Polymerase	AJ279824/CAC19161	[49]
*Trichoplusia ni ascovirus 2a*	Trichoplusia ni ascovirus 2a	TnAV-2a	pol for DNA Polymerase	AJ279826/CAC19180	[49]
			Major capsid protein (MCP)	AJ312707/CAC84132	[38]
	Trichoplusia ni ascovirus 2b	TnAV-2b	po1 for DNA Polymerase	AJ279827/CAC19181	[49]
	Trichoplusia ni ascovirus 2d (2005)	TnAV-2d	po1 for DNA Polymerase	DQ015959/AAY43139	[33]
			Major capsid protein (MCP)	DQ015960AAY43140	
*Heliothis virescens* *ascovirus 3a*	Heliothis virescens ascovirus 3a	HvAV-3a	po1 for DNA Polymerase	AJ279817/CAC19156	[49]
	Heliothis virescens ascovirus 3b	HvAV-3b	po1 for DNA Polymerase	AJ279818/CAC19157	[49]
	Heliothis virescens ascovirus 3c	HvAV-3c	po1 for DNA Polymerase	AJ312696/CAC84471	[38]
			Major capsid protein (MCP)	AJ312704/CAC84482	
	Heliothis virescens ascovirus 3d	HvAV-3d	po1 for DNA Polymerase	AJ279820/CAC19159	[49]
	Heliothis virescens ascovirus 3e	HvAV-3e	po1 for DNA Polymerase	EF133465/ABO37187	[4]
			Major capsid protein (MCP)	EF133465/A4KXB1	
	Heliothis virescens ascovirus 3f (2005)	HvAV-3f	po1 for DNA Polymerase	DQ015956/AAY43136	[33]
			Major capsid protein (MCP)	AF419098/AAL16646	
	Heliothis virescens ascovirus 3g (2000)	HvAV-3g	po1 for DNA Polymerase	AJ620611/CAF05813	[50]
			Major capsid protein (MCP)	AJ620613/CAF05815	
	Heliothis virescens ascovirus 3h	HvAV-3h	po1 for DNA Polymerase	JQ706081	This study
			Major capsid protein (MCP)	JQ706080	This study

Analyses of evolutionary divergence between sequences were conducted in MEGA5 and the number of base differences per site from analysis between sequences is shown in Table 2. All results were based on the pairwise analysis of 11 sequences, including one outgroup and ten comparative isolates. All of aligned 780 amino acid positions were eliminated from the data set. The genetic distances of the isolates of HvAV-3a for combined protein sequences ranged from 0.012 (HvAV-3f vs. HvAV-3g) to 0.051 (HvAV-3c vs. HvAV-3g) (Table 2). The largest distance of HvAV-3h with the isolates of HvAV*-3a* is 0.043 (HvAV-3c vs. HvAV-3h), which is smaller than 0.051 (HvAV-3c vs. HvAV-3g). The p-distances analysis provides support that HvAV-3h isolated from *S. exigua* is one variant of the species HvAV-3a.

**Table 2:** Uncorrected pairwise p-distances among the combined protein data of ascoviruses with the outgroup CIV.

	1	2	3	4	5	6	7	8	9	10
1 CIV										
2 DpAV-4a	0.634									
3 TnAV-6a	0.691	0.735								
4 SfAV-1a	0.714	0.735	0.623							
5 TnAV-2a	0.691	0.739	0.636	0.319						
6 TnAV-2d	0.689	0.743	0.638	0.313	0.033					
7 HvAV-3c	0.695	0.743	0.640	0.325	0.025	0.041				
8 HvAV-3e	0.693	0.737	0.636	0.311	0.031	0.010	0.039			
9 HvAV-3f	0.691	0.739	0.632	0.313	0.008	0.025	0.016	0.023		
10 HvAV-3g	0.695	0.739	0.636	0.319	0.043	0.023	**0.051**	0.012	0.035	
11 HvAV-3h	0.698	0.737	0.640	0.317	0.039	0.023	0.043	0.016	0.031	0.029

The number in bold is the largest distances between the isolates in the same species.

## Discussion

Among all ascovirus species discovered in America, the HvAV-3a species is the only ascovirus widely distributed in other areas including Australia, Indonesia and China. From the phylogenetic analysis in this study, HvAV-3h has a close relationship with HvAV-3g isolated from *S. exigua* in Indonesia, and HvAV-3g is closely related to HvAV-3e from Australia. Although HvAV-3a was first isolated from its host *H. virescens* in the USA, it has a wide host-range and can infect *S. exigua* and *S. litura* which have origins in Asia [25]. This data suggests that the geographical origin of the species HvAV-3a may be in Asia. This data also supports the notion that the primary host of HvAV-3a is *S. exigua*. The US found HvAV-3a and other HvAV-3a variants may have been introduced to North America when *S. exigua* was introduced into and established in Oregon in 1876 [25]. To know more on the origin of ascoviruses and their association with the host insects, additional ascovirus isolates from Asia, especially from China, and more sequence data for phylogenetic analysis are necessary.

One concern of the HvAV-3h used in this study is the possible contamination of additional viruses such as other ascoviruses or baculoviruses because it was field-isolated. However, TEM examination of the hemolymph of the field-isolated HvAV-3h, cell infection (Fig. 5) and PCR detection for *S. exigua* multicapsid nucleopolyhedrovirus (SeMNPV) and *Helicoverpa armigera* SNPV (HearSNPV) commonly found in Hunan province in the HvAV-3h inoculum failed to detect baculovirus contamination (Figure S2).

The most dramatic cytopathic effect of ascovirus infection in insect cells is its ability to induce apoptosis that leads to the formation of vesicles to enclose virions [2]. This cytopathic effect is thought to be caused by the viral caspase of SfAV-1a [22]. HvAV-3e has a caspase-like gene but does not induce apoptosis in Sf21 when the HvAV-3e caspase gene is stably expressed in Sf9 cells. However, HvAV-3e infection in HzFB cells showed significant cell distortion typical of ascovirus cell infection [26]. Similar cytopathology of HvAV-3g infection in Sf21 cells was also observed [27]. During *in vitro* infection of HvAV-3h, some SeE1 and Sf9 cells showed blebbing of the cytoplasmic membrane which leads to the production of vesicles. This *in vitro* response is analogous in an *in vivo* infection due to the vesicle production in the host *S. exigua* larvae (Fig. 1D, Fig. 5A-36 and A-48, Fig. 5 B-36 and B-48). The mechanism of cytoplasmic membrane blebbing induced by HvAV-3h is unknown. Also whether there were any HvAV-3h virions in these vesicles is also unknown but is currently under investigation. The absence of cell cleavage of HvAV-3h is also unclear. It may be too early to conclude that HvAV-3h does not have a caspase gene since the genome sequence is currently unavailable. It is likely that HvAV-3h, like HvAV-3e, has a caspase gene since it induces *S. exigua* larval cell apoptosis for the production of vesicles (Fig. 1D). One possible explanation for the different abilities of HvAV-3h to cleave the cells for the production of vesicles involves cellular proteins that regulate the HvAV-3h induced apoptosis.

Different cytopathological effects of insect cells have been observed during HvAV-3e replication in Sf9 cells, HzAM1 cells (derived from *Helicoverpa zea)* and FB33 cells (derived from the *H. zea* fat body), in addition to cell deformation to no symptoms in *Pieris rapae* cells [28]. However, no actual DNA replication assay was performed. We compared the DNA replication kinetics of HvAV-3h during infection of SeE1 and Sf9 cells. It is unknown how ascovirus replicates in insect cells but Xue and Cheng suggest that ascovirus may use a rolling circle replication mechanism similar to baculovirus and herpes simplex virus due to the presence of a hypervariable region in the genome of SfAV-1d [29], [30], [31]. This hypothesis still requires testing.

## Materials and Methods

### Insects, Insect Cells, Viruses and Viral DNA

Insects used in this report included three species, *S. exigua*, *H. armigera*, *S. litura*. Except *H. armigera* that was provided by Dr. Xuelian Sun of Wuhan Institute of Virology, the other two species were collected from cotton fields. Insects were reared on artificial diets in incubators with temperature at 27±1°C, relative humidity 70-80% and photoperiod (L/D, 16∶8). Insect cells included SeE1 received from Dr. A. H. McIntosh of Biological Control of Insects Research Laboratory, Missouri [32] and maintained in HyQ® SFX-Insect MP™ (HyClone, Utah) serum free media supplemented with 5% fetal bovine serum [32] and Sf9 purchased from Invitrogen (Carlsbad, California) and maintained in TNM-FH supplemented with 10% fetal bovine serum. Insect cells were cultured in an incubator with temperature controlled at 27±0.5°C. Viral DNA included *Autographa californica multicapsid nucleopolyhedrovirus* (AcMNPV) E2, SfAV-1a, HvAV-3g, HvAV-3f and TnAV-2d prepared previously in the lab [27], [33]. HvAV-3h virions were purified from hymolymph of *S. exigua* larvae infected with HvAV-3h in this report.

### Ascovirus Isolation

Insect collections were conducted from June to October, 2011 in a university research experimental cotton field that does not require specific permits for the described field studies in Donghu Town, Changsha City, Hunan Province,China. The cotton field was sprayed with insecticides on a weekly basis. The larvae of *S. exigua* on cotton plants were collected by hand and were placed individually in diet tubes and brought to the laboratory for observation. Cotton leaf damages caused by *S. exigua* larvae were recorded by photographing. Individual larvae showing retarded larval development suggesting ascovirus infection were examined by hemolymph sampling for color changes and the evidence of vesicle formation following the reported methods [3], [27]. Hemolymph color of healthy and virus-infected larvae was recorded by photographing. Hemolymph of a single *S. exigua* larva labeled number 2 was used to inoculate healthy third instar larvae of *S. exigua* for collecting enough hemolymph to perform DNA analyses and cell infection studies.

### Identification of Ascovirus by Transmission Electron Microscopy

The hemolymph of infected *S. exigua* larvae was harvested by centrifugation at 500 × g for 5 min. The pellets were processed for TEM [34]. Examination of hemolymph for viruses was conducted using a Joel JEM-1200EXII TEM. Samples showing viruses were photographed with a digital camera attached to the TEM. The sizes of the virions were measured.

### DNA Dot Blot, Restriction Profile and Southern Hybridization Analysis

Hemolymph of HvAV-3h infected larvae at day 14 p. i. was collected for virion purification and DNA extraction following the reported protocol [27]. In dot blot DNA-DNA hybridization, 100 ng each purified genomic DNA of AcMNPV (negative control), HvAV-3h, HvAV-3g, HvAV-3f, SfAV-1a and TnAV-2d were blotted to a Magnaprobe nylon membrane (OSMONICS Inc., Minnesota) using a S&S Minifold-I (Schleicher & Schuell BioScience, New Hampshire) following recommended protocols provided by the manufacturers. Each DNA was blotted in triplicate. The blot was hybridized with a probe of HvAV-3h DNA digested by *Sau*3A I and labeled with biotin using a NEBlot™ Phototope kit (NEB) following the protocol of the kit manufacturer. The hybridization conditions followed reported conditions [27]. A Streptavidin/Biotinylated alkaline phosphatase detection system (NEB) was used for the detection of hybridization. RFLP and Southern hybridization analysis followed the reported protocol [34]. Purified viral genomic DNA (400 ng each) of AcMNPV, HvAV-3h, HvAV-3g, HvAV-3f, SfAV-1a and TnAV-2d was individually digested with *Eco*R1 or *Hin*dIII and analyzed by 0.7% agarose gel electrophoresis (45V/cm for 14 hours) and stained with ethidium bromide for documentation. The DNA on the agarose gel was then transferred to a Magnaprobe nylon membrane and hybridized with the HvAV-3h probe as prepared for dot blot and hybridization was detection as descripted for dot blot [27], [31].

### Cell Infection and DNA Replication Kinetics

An *Eco*R1 restriction library was constructed according to Cheng et al. [35]. One clone (E07) was sequenced and the obtained sequence (accession number: JQ706079) was used to design a pair of real-time qPCR primers for HvAV-3h titer estimation. HvAV-3h virions were extracted from hemolymph of *S. exigua* larvae infected with HvAV-3h. An aliquot of HvAV-3h was used for viral DNA extraction. The HvAV-3h DNA with known concentration in the DNA hybridization studies was serially diluted to be used as template in the construction of a standard curve by the real-time qPCR method using an iQ SYBR Green Supermix kit (BIO-RAD, Hercules, USA), following the recommended conditions of the kit manufacturer, with a BIO-RAD ICycler iQ™ system using a primer pair (AV7-F: 5'-GCGATAGATATCCGCAGGAA-3'; AV7-R: 5'-AAACATGTTCGATGCAGCAC-3') to estimate DNA copy number of HvAV-3h virion or virion titer according to Lo and Chao [36]. Conversion of DNA weight to DNA copy number is based on the formula (assumption) that each mole of a DNA base pair weighs 650 gram. The HvAV-3h titer estimated by viral DNA copy number was further confirmed with the end-point dilution method [37].

Sf9 and SeEI cell lines were seeded in six-well plates at 5×10^5^ cells/well, for 1 h. After attachment, both cells were infected with the same inoculum of HvAV-3h virion at a multiplicity of infection (MOI) of 170 virions/cell for 1 h (viral absorption). The HvAV-3h inoculum was removed and the cells were refreshed with 2 ml of respective cell growth media and incubated in an incubator at 27°C. Control cells were mock infected with respective cell growth medium. Cell infection was monitored every 12 h p.i. by microscopy and 4 random images were taken with an attached digital camera until the experiment was completed at 240 h p. i. Cell infection rates relative to mock infection were calculated by dividing the number of cells showing viral infection (cell blebbing and deformation) by the total number of cell in the picture. The two cell infections were compared by regression analysis using Excel (Microsoft).

To investigate the dose-response of Sf9 and SeE1 cells to HvAV-3h infection, the HvAV-3h stock was serially diluted 10-fold with respective media to infect the two cell lines as described above. Therefore, in this infection, the cells were infected at MOIs of 170, 17, 1.7 and 0.17. The two cell lines were also mock infected to serve as a control. Data collection followed what described above. The dose-response was analyzed using Excel (Microsoft).

To compare the early DNA replication kinetics of HvAV-3h in the two cell lines, Sf9 and SeE1 cells were infected in triplicate with HvAV-3h, as described above. Cells were harvested at 0, 6, 12, 24, 36, 48 h p.i. by dislodging and centrifugation at 500 ×g for 5 min to pellet the cells. The pelleted cells were stored at -20°C until the experiment was completed at 48 h p.i. The Sf9 and SeE1 cell pellets stored at -20°C were used for DNA extraction to estimate HvAV-3h genome copy numbers based on the standard curve construction running simultaneously with the viral DNA replication samples using primer pair AV7-F and AV7-R by real-time qPCR [47]. The replication kinetics of HvAV-3h in the two insect cell lines was compared by regression analysis using Excel (Microsoft).

### Host Range

To understand the host range of HvAV-3h, important local noctuid pests, *H. armigera*, *S. exigua* and *S. litura* were tested. For each species, 30 third-instar larvae in each of the three replicates were inoculated with HvAV-3h from the hemolymph of *S. exigua* larvae infected with HvAV-3h as described [27]. Control larvae were injected with the hemolymph from healthy larvae. Treated and control larvae were reared on artificial diets in an incubator at 27±1°C and 70-80% humidity. Mortality was checked daily until pupation of some of the larvae. Corrected mortality of each species was analyzed for variance (ANOVA) to compare the difference among the mortality of three tested species by the SAS program.

### Phylogenetic Analysis

The major capsid protein (MCP) gene of HvAV-3h was PCR-amplified with previously published primers specific to ascovirus MCP (Forward: 5′-GGG AAT TCC AAT GAC TTC AAA CCC AGA AAC-3′; Reverse: 5′-GCG GAT CCT CAT TGA AAT CGC CTC CGT TGT-3′) [38], and the HvAV-3h DNA polymerase gene was amplified with a new set of primers specific to the DNA polymerase (HAV3-pol-F: 5′-CCAGGATCACCAACACAC-3′; HAV3-pol-R: 5′-GCTAGAGGATCGCTAACG-3′) using the high fidelity *pfu* DNA polymerase (Clontech Laboratories Inc., California) with HvAV-3h DNA as template. The PCR products were separated by agarose gel electrophoresis and purified by the GENECLEAN® Kits (Bio101) for direct sequencing using BigDye Terminator v3.1 cycle sequencing kit (Applied Biosystem, California) with the reverse primer for each gene by PCR and analyzed with an ABI 3730 DNA Analyzer. The obtained nucleotide sequences were translated into protein sequences using Molecular Evolutionary Genetics Analysis software version 5.0 (MEGA 5.0) [39]. The protein sequences data of the two genes of all the known ascovirid species including all the isolates listed [19] were downloaded from the National Center for Biotechnology Information database. Based on a previous study, Chilo iridescent virus (CIV) type 6 was selected as the out-group for phylogenetic analysis [19]. All the protein sequences data were collected through importing into MEGA 5.0 and aligned by using Clustal X 2.0 [40], and the data of those two genes were combined. Prottest 3.63 program was used to select the best-fit model for the combined amino acid substitution [41]. A maximum-likelihood tree and MrBayes tree were constructed by MEGA 5.0 and MrBayes 3.1 based on the selected best-fit model, respectively [39], [42], [43]. Finally, the trees were shown through treeviewer X [44]. The MP (Maximum parsimony) and NJ (Neighbor Joining) phylogenetic trees were inferred from aligned matrix using PAUP 4.0 analyses [45], and Bootstrap analyses were carried out based on a full heuristic search of 10,000 pseudo replicates [46].

## Supporting Information

Figure S1 **Comparison of cell infection of HvAV-3h at 108 h post infection.** A, uninfected SeE1 cells. B, SeE1 cells infected with HvAV-3h showing limited vesicle production with most of cells killed but not cleaved into vesicles. C, uninfected Sf9 cells. D, Sf9 cells infected with HvAV-3h showing more vesicle production than SeE1(B) with most of the Sf9 cells cleaved. Scale bars, 50 µm.
(TIF)

Figure S2 **PCR examination of HvAV-3h for baculovirus contamination.** NTC, no template control. DNA polymerase (pol) primers: HAV3-pol-F: 5′-CCAGGATCACCAACACAC-3′; HAV3-pol-R: 5′-GCTAGAGGATCGCTAACG-3′. SeMNPV fp25k primer: SeFP25k-F 5'-ACA TGT TGT CGT GCG GC-3' SeFP25k-R 5'-GAG GAA ACA TCG CTC ACA C-3'. HearSNPV fp25k primers: Hafp25k-F, 5'-CCA TAT TTG GTG ACC GC-3', Hafp25k-R 5'-CGG TAC TCG GTA AAT CTG-3'.
(TIF)
