# Supplementary figures and images for: Phylogenetic Position and Replication Kinetics of Heliothis virescens Ascovirus 3h (HvAV-3h) Isolated from Spodoptera exigua
Source: PLoS One. 2012 Jul 5;7(7):e40225. doi: 10.1371/journal.pone.0040225 (PMC3390325; doi:10.1371/journal.pone.0040225)

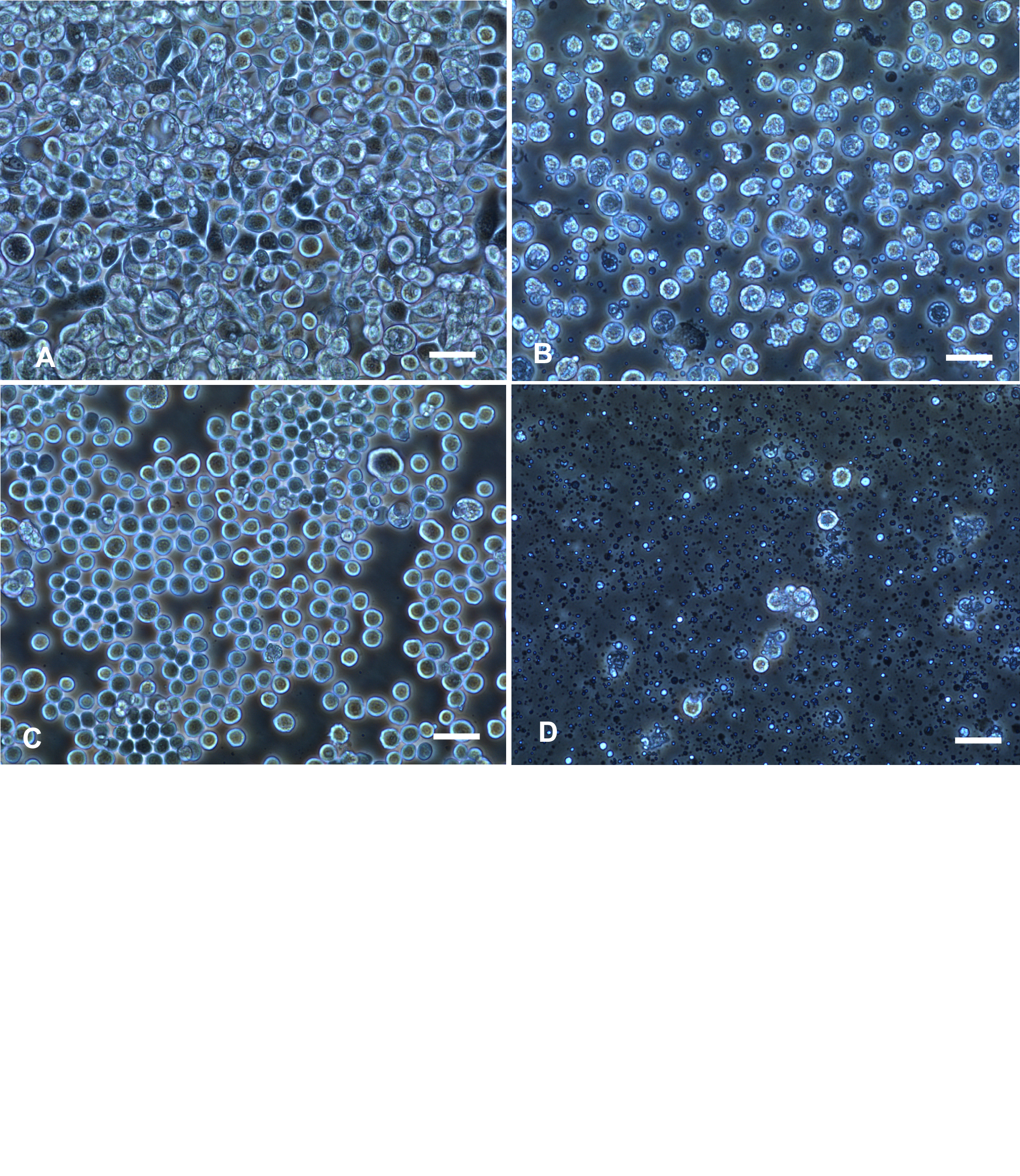

Supplement: Figure S1 — Comparison of cell infection of HvAV-3h at 108 h post infection. A, uninfected SeE1 cells. B, SeE1 cells infected with HvAV-3h showing limited vesicle production with most of cells killed but not cleaved into vesicles. C, uninfected Sf9 cells. D, Sf9 cells infected with HvAV-3h showing more vesicle production than SeE1(B) with most of the Sf9 cells cleaved. Scale bars, 50 µm. (TIF) [file pone.0040225.s001.tif]

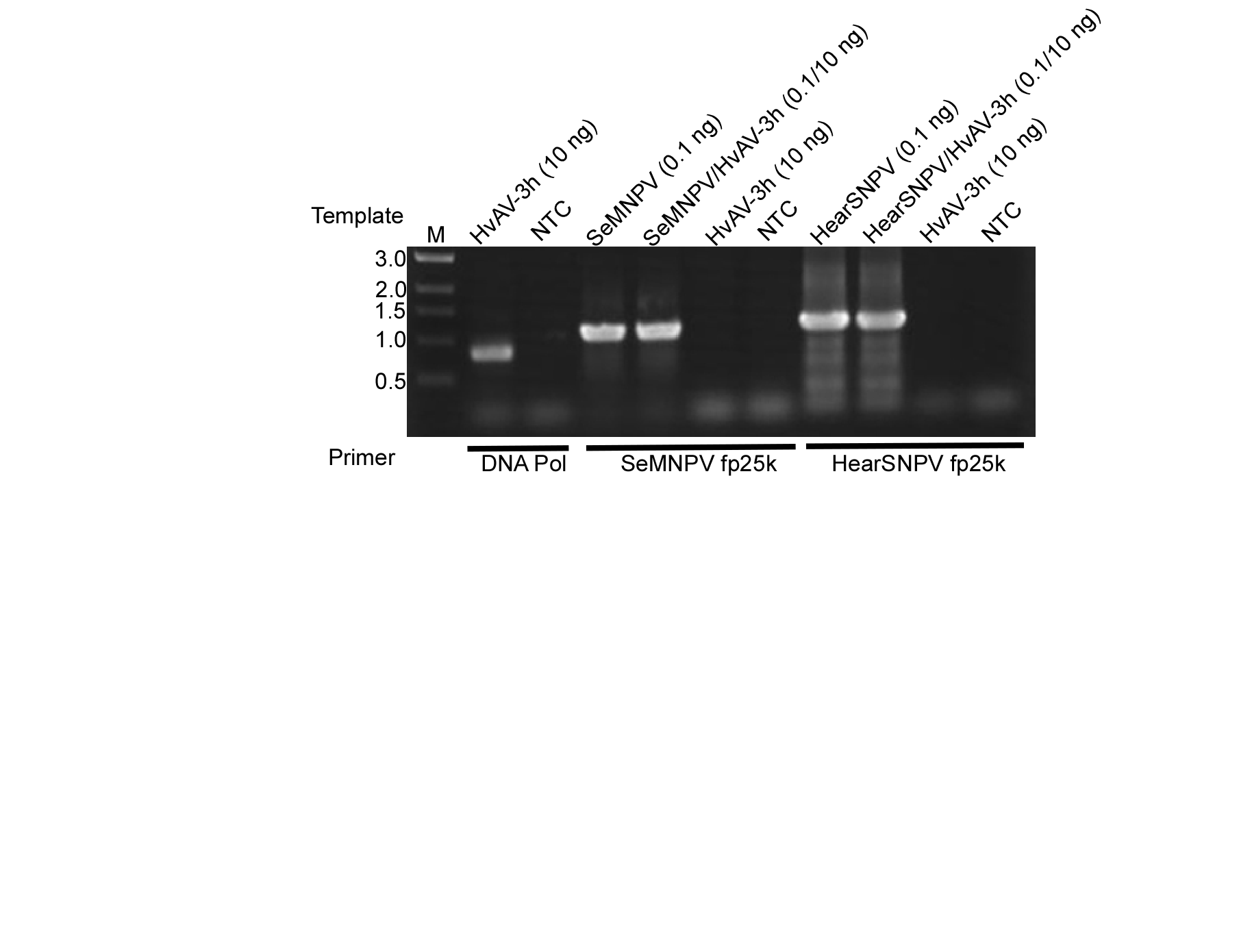

Supplement: Figure S2 — PCR examination of HvAV-3h for baculovirus contamination. NTC, no template control. DNA polymerase (pol) primers: HAV3-pol-F: 5′-CCAGGATCACCAACACAC-3′; HAV3-pol-R: 5′-GCTAGAGGATCGCTAACG-3′. SeMNPV fp25k primer: SeFP25k-F 5'-ACA TGT TGT CGT GCG GC-3' SeFP25k-R 5'-GAG GAA ACA TCG CTC ACA C-3'. HearSNPV fp25k primers: Hafp25k-F, 5'-CCA TAT TTG GTG ACC GC-3', Hafp25k-R 5'-CGG TAC TCG GTA AAT CTG-3'. (TIF) [file pone.0040225.s002.tif]
